# Dietary supplementation with radionuclide free food improves children's health following community exposure to ^137^Cesium: a prospective study

**DOI:** 10.1186/s12940-015-0084-x

**Published:** 2015-12-22

**Authors:** Daria M. McMahon, Vitaliy Y. Vdovenko, Yevgenia I. Stepanova, Wilfried Karmaus, Hongmei Zhang, Euridice Irving, Erik R. Svendsen

**Affiliations:** Department of Epidemiology and Biostatistics, Arnold School of Public Health, University of South Carolina, 915 Greene Street, Columbia, SC 29208 USA; Research Center for Radiation Medicine, Academy of Medical Sciences of Ukraine, 53 Melnikova St., Kiev, 04050 Ukraine; Division of Epidemiology, Biostatistics, and Environmental Health Science, School of Public Health University of Memphis, 301 Robison Hall, Memphis, TN 38152 USA; Tulane University School of Public Health and Tropical Medicine, 1440 Canal Street, New Orleans, LA 70112 USA; Department of Public Health Sciences, Medical University of South Carolina, 135 Cannon Street, Suite 303, Charleston, South Carolina 29425 USA

**Keywords:** Ionizing radiation, Chernobyl, Blood indices, Regression discontinuity

## Abstract

**Background:**

Following the Chernobyl nuclear disaster of 1986, vast areas of Ukraine became contaminated with radionuclides. We examined health effects of school-based food intervention for children in a rural region Narodichi, Ukraine, exposed to low-level radiation through diet of locally produced foods. Until 1995, children received three daily meals with low content of artificial radionuclides which were subsequently reduced to two.

**Methods:**

Annual health screening data (1993–1998) were examined using a quasi-experimental regression discontinuity analysis (*n* = 947 children; 3,573 repeated measurements). Generalized Estimating Equation models evaluated effect of the food supplementation reduction on hematologic measures and prevalence of anemia, acute respiratory illnesses and diseases of immune system.

**Results:**

Prior improvement of several hematologic parameters diminished after food supplementation was reduced. From 1995 to 1996, levels of hemoglobin and erythrocytes decreased from 12.63 (95 % CI: 12.56-12.71) to 12.46 g/dL (% CI: 12.39-12.52) and from 4.10 (95 % CI: 4.07-4.12) to 4.02 (95 % CI: 4.00-4.04) × 10^12^/L, respectively. In agreement, the prevalence ratio (PR) of previously declining anemia increased from 0.57 to 1.31 per year (p_interaction_ < .0001). The relation between food supplementation and hemoglobin levels was modified by residential ^137^Cs soil levels. After food supply reduction, PR of common cold and bronchitis increased from 1.27 to 2.32 per year (p_interaction_ = 0.01) and from 1.09 to 1.24 per year (p_interaction_ = 0.43), respectively.

**Conclusions:**

Food supplementation provided by the Ukrainian government likely prevented development of anemia in many of the children residing in the contaminated district. Food supplementation after the community exposure to radioactivity through a diet of locally grown foods should be considered as an effective approach to reduce adverse health effects of radiation.

**Electronic supplementary material:**

The online version of this article (doi:10.1186/s12940-015-0084-x) contains supplementary material, which is available to authorized users.

## Background

Immediately after the Chernobyl nuclear disaster that occurred on April 26, 1986, the environment within the region of the nuclear power plant was contaminated with different gamma-emitting radionuclides including ^131^I, ^134^Cs, ^137^Cs and with beta-emitter ^90^Sr [1]. The predominant long-term radionuclide has been Cesium-137 (^137^Cs) with the half-life of 30 years. After the Chernobyl disaster, the Ukrainian government conducted environmental monitoring of ^137^Cs soil contamination, and the results were published in several reports by the Ministry of Health and the Ministry of Emergency Situations of Ukraine [2, 3]. Even the most recent reports still show contamination of regional soil and locally produced cow milk and potatoes with radionuclides, although to a lesser extent than what was previously observed [4, 5]. The greatest public health concern has been the potential for bioaccumulation of ^137^Cs in locally grown foods and forest products such as mushrooms, berries and game which may cause adverse health effects in their consumers [6]. For decades, transfer of radionuclides from the environment through the food chain to humans has been the major pathway of the exposure to chronic low-dose radiation [7]. Cesium-137 is taken up by crops and forest products through their roots and is easily transported to the leaves, flowers, nuts, and fruits [8–10]. The transfer of ^137^Cs into vegetation is strongly influenced by the type of soil and is the highest in Ukrainian and Belarusian Polyssia, a large agricultural area in northern Ukraine and southern Belarus located in close proximity to Chernobyl, where the predominant soil type is marsh and peat [11]. Further along the food chain, contamination of meat is mainly attributed to contaminated soil, grass, nuts and mushrooms eaten by livestock. In the Ukrainian Polyssia, 70-75 % of daily ^137^Cs intake is considered to come from milk and dairy products, 5-10 % from meat, 10-15 % from mushrooms and berries, and 10-15 % from potatoes and vegetables [11]. Levels of radionuclides in humans seem to vary with seasons, likely due to the variation of availability of contaminated food [7, 12, 13]. Although wild berries and mushrooms contain high quantities of ^137^Cs, their consumption is limited to the summer and fall, while milk is available throughout the entire year in all areas and widely consumed by both children and adults [11, 14]. Levels of ^137^Cs in milk vary between different areas [2, 4, 5, 14]. Communities living in the contaminated territories have been, thus, chronically and continuously exposed to radiation by eating contaminated locally produced or harvested foodstuffs [12]. Throughout all post-incident years, consumption of radionuclide-contaminated local produce contributed at least 50 % of a total individual absorbed dose [15]. As the external radiation exposure decreases over time, the contribution of the internal dose to the total dose increases [16]. It is considered that children are more sensitive to ionizing radiation than adults [17]. In children, a larger surface-to-volume ratio and a higher metabolic rate due to growth and development leads to a higher need for calories and more food consumed per kilogram of bodyweight than in adults [18]. In addition, it has been indicated that the accumulation of ^137^Cs in organs of children is higher than in adults [19, 20]. Therefore, the Ukrainian government implemented public health interventions aimed to reduce the exposure potentially received by the public due to the consumption of locally produced contaminated food. After the Chernobyl radiological disaster occurred in 1986, government officials began a public health intervention to reduce radiation exposure in children. Every child in the agricultural Narodichi Region located in the Ukrainian Polyssia was provided three complete meals of uncontaminated food each day at school until the program funding was reduced in 1995 and only two meals were provided each day. Little is known quantitatively about the effectiveness of public health strategies such as these to prevent adverse health outcomes following a community-wide radiological exposure event.

In this study we assessed the beneficial health effects of a school-based public health intervention on a pediatric population chronically exposed to radiation through foods grown within a region of Ukraine contaminated by radiation after the Chernobyl disaster. Herein we compared different measures of health status, including blood indices and the prevalence of different health conditions in these children both before and after the public health intervention was reduced using a quasi-experimental regression discontinuity design.

## Methods

### Study population

The Narodichi region is a peasant farming region located about 80 km from the Chernobyl nuclear site to the west of the evacuation zone and is one of the areas with the highest ^137^Cs soil contamination levels in Ukraine [3]. Most of the population lives in rural villages and small towns. The exact number of children residing in this area is unknown, but according to the latest All-Ukrainian Population Census, in 2001 it was about 2,000 [21]. As part of public health interventions aimed at the protection of the population from exposure to environmental radiation, annual medical examinations were mandated for all children living in the contaminated areas each spring from 1986 to 2006. This quasi-experimental regression discontinuity study examines the public health surveillance data from the annual medical screenings of 947 school children (6–16 years old) participants of the Narodichi Children Cohort (NCC) [22, 23] who provided blood samples. The NCC is a dynamic cohort where new children entered and the older ones left the cohort once they turned 19 years of age; thus, not all children participated in all years. From 1993 through 1998, the follow-up of 947 children with blood samples yielded 3,573 repeated measurements.

### Exposure, hematologic measurements, and diagnostics

In previous research within this ^137^Cs exposed population an adverse effect of the level of soil ^137^Cs contamination was found on hematologic parameters [22]. This investigation also used the average levels of ^137^Cs soil contamination in the villages as a proxy for individual exposures. Children resided in 34 different settlements of the Narodichi region. All villages and towns had ^137^Cs soil measurements taken in 1992 with concentrations ranging from 51 to 356 kBq/m^2^. For all consecutive years (1993–1998), the average levels of ^137^Cs soil contamination were calculated based on the 1992 measurement and a decay function and are publically available in reports by the Ministry of Health and the Ministry of the Emergency Situations of Ukraine [2, 3]. The proxy of the individual exposure to ^137^Cs (external and internal) used in this study represents the interquartile range (199 kBq/m^2^) normalized average ^137^Cs soil concentration at each child’s village of residence. The interquartile range was determined for ^137^Cs soil concentrations in all villages and for all years of observations combined. The individual whole body content of ^137^Cs were measured in Bq using a gamma-spectrometer (Whole-Body Counter SCRINNER-3 M, designed and produced by INECO (Ukrainian Institution of Human Ecology, Academy of Technological Sciences)) as was described elsewhere [6]. However, these data were not available for all children. Calculations of the individual whole body content of ^137^Cs used calibration coefficients to account for child's weight. To further reduce a chance of confounding by weight, we additionally adjusted the individual whole body content of ^137^Cs for child's weight (Note: whole body content of ^137^Cs was divided by child's weight, Bq/kg). For the hematologic parameters, blood sampling data collected during the mandatory clinical examinations were used. Blood was collected in EDTA tubes and blood count analysis conducted using Sysmex model F-800. Blood smears were stained according to the standardized GIEMSA procedure. A novel sensitive biomarkers of chronic inflammation, the platelet/lymphocyte and neutrophil/lymphocyte ratios [24], were calculated using complete white blood cell count. Concentrations of immunoglobulins (Ig) A, G, and M were determined in fasting serum samples collected in EDTA tubes using reverse radial immunodiffusion (Mancini technique). At the yearly health examinations, all children were examined by visiting pediatricians from the Research Center of Radiation Medicine. All health conditions were diagnosed according to the International Classification of Diseases, Ninth Edition (ICD-9).

### Public Health Intervention

To reduce the dietary exposure to ^137^Cs, every child was provided three meals with low content of artificial radionuclides each day at school from 1986 until the program funding was decreased in 1995 and then only 2 meals each day were provided. The meals were prepared to meet nutritional standards determined by the Ukrainian Government for the appropriate age groups [25]. The effect of the reduction of this food supplementation on the hematologic parameters and occurrence of various health conditions was examined herein by contrasting those measures during the highest intervention years (1993–1995) with the reduced-intervention years (1996–1998) using regression discontinuity design.

### Statistical approach

In principal, the Chernobyl accident resulted in a natural experiment, since radiation was distributed independently from potential confounding variables. There were six annual measurements of the hematologic parameters between 1993 and 1998. To assess the effectiveness of the school-based food intervention which was reduced after the 1995 school year, we used a regression discontinuity analysis [26]. This type of analysis is applicable in quasi-experimental studies where treatment is assigned based on the threshold rule and the study subjects cannot manipulate the assignment. At the threshold, the study participants have an equal chance to be either in the treatment or control group and are similar in all characteristics except the exposure. Thus, the treatment assignment near the threshold works similar to randomization in clinical trials, which eliminates residual confounding and allows for causal inferences [27]. In this study, the assignment variable is the yearly period of time from 1993 through 1998 when six yearly health examinations were conducted. The treatment is the number of meals with low content of artificial radionuclides; it was assigned based on the threshold assignment rule. The supply of radiation-free food was financed by Ukrainian governmental program. Throughout the end of the 1995 school year (May-June), children at school received three meals per day. After the summer break, in September 1995, the number of meals was reduced to two per day at the same time for all children due to the reduction of funding. Thus, statistically, a "sharp" type of the regression discontinuity design is appropriate for this study [28], and the threshold is mid-year 1995. Under the above mentioned circumstances children could not manipulate the treatment which is another feasibility criterion for the regression discontinuity design [28]. Since in this cohort children's age increased and the levels of residential ^137^Cs in the soil decreased overtime, these two variables were not balanced near the threshold (mid-year 1995); therefore, we included them as covariates in the statistical models. Since the objective of the study was to compare changes in blood indices and disease prevalence before and after the food supplementation reduction, the study period was divided into two time periods: 1993–1995 and 1996–1998. The temporal changes in different blood cell counts and the hemoglobin level during two different periods of food intervention were estimated using linear models with repeated measures (proc genmod in SAS) (time trends 1993–1995 vs. 1996–1998). Generalized Estimating Equations (GEE) with an exchangeable correlation structure were used to estimate the parameters [29]. This statistical method infers an average response over the population and takes into account the between-subjects effect to prevent overestimation of standard errors for the time-dependent predictors due to repeated measurements [30]. The independent variables in the statistical models included time (categorical, represents temporal order of each clinical examination from 1 to 6), food intervention status (2 meals/day vs. 3 meals/day), gender, age (continuous), interquartile range normalized annual village soil ^137^Cs levels, and an interaction term between food intervention status and time (food × time). Since yearly physical examinations were always conducted in February-April, the season of the year was not a confounder and, therefore, was not included in the statistical models. For the statistical analyses, basophils, eosinophils, leukocytes, lymphocytes, monocytes, neutrophils, platelets, platelet/lymphocyte ratio and neutrophil/lymphocyte ratio were log-transformed, and the results were back-transformed to yield geometric means.

We examined the effect modifying role of residential ^137^Cs soil contamination in the relationships between the food intervention and blood hemoglobin levels using stratification by quintiles of soil ^137^Cs (51–106, 107–127, 128–218, 219–318 and 319–356 kBq/m^2^). The statistical models included time (categorical), food intervention (2 meals/day vs. 3 meals/day), age, gender, and food × time interaction.

To calculate prevalence ratios of anemia, diseases of respiratory and immune systems we used log-binomial models with repeated measures to infer serial correlation [31]. The covariates in the model included time (categorical), food intervention (2 meals/day vs. 3 meals/day), age, gender, interquartile range normalized annual village soil ^137^Cs levels, and food × time interaction. All data analyses were performed using SAS software (Version 9.3: Statistical Analysis System, Cary, NC) with significance level α = 0.05.

## Results

Although the pediatric health screening was mandatory, it was not enforced. Approximately 75 % of the children in the region participated in the screenings annually. In total 1,459 children were screened and included in the NCC. Of these, 1,247 provided blood samples (86 %). Of all, 947 children were 7–16 years old and included in this analysis. A total of 3,573 repeated blood measurements were available over the 6 years of observation. The characteristics of the study population are described in Table 1. The individual whole body content of ^137^Cs adjusted for body weight (Bq/kg) increased between 1993 and 1998 (Table 2) and was correlated with residential ^137^Cs soil levels (Bq/m^2^) (Spearman r = 0.26, *p* < .0001). Except for erythrocyte count and hemoglobin concentration, none of the hematologic markers were correlated with one another. The interclass-correlation coefficients over the total time period of 6 years averaged around 0.50, an indication of reasonable stability of the measurements.

**Table 1:** Characteristics of the study population

	Children participating (*n* = 947) n (%)	Total number of observations (*n* = 3,573) n (%)
Gender		
Male	454 (48)	1,674 (47)
Age at enrollment		
7-11	795 (84)	3,053 (85)
12-15	133 (14)	500 (14)
≥16- < 17	19 (2)	20 (0.6)
Age at the examination, years	mean ± std 11.2 ± 2.6	
Year of birth		
1979	1 (0.1)	2 (0.1)
1980	57 (6)	205 (6)
1981	61 (6)	267 (7)
1982	102 (11)	482 (13)
1983	121 (13)	566 (16)
1984	114 (12)	588 (16)
1985	90 (9)	457 (13)
^a^	-	-
1987	83 (9)	331 (9)
1988	101 (11)	296 (8)
1989	103 (11)	217 (6)
1990	67 (7)	115 (3)
1991	47 (5)	47 (1)
Year of first participation		
1993	484 (51)	2,471 (69)
1994	76 (8)	322 (9)
1995	77 (8)	267 (7)
1996	56 (6)	148 (4)
1997	190 (20)	301 (8)
1998	64 (7)	64 (2)
Quintiles of the area contamination: ^137^Cesium (kBq/m^2^)		
51-104	158 (17)	580 (16)
105-127	193 (20)	699 (19)
128-219	221 (23)	852 (24)
220-318	165 (17)	492 (14)
319-356	210 (22)	950 (27)

std: standard deviation

^a^Children born in 1986 were analyzed and reported in a separate paper [40]

**Table 2:** Means of weight-adjusted whole body content of Cesium-137 by year

Weight-adjusted ^137^Cs whole body content, Bq/kg	3 meals/day			2 meals/day		
	1993	1994	1995	1996	1997	1998
	*n* = 477	*n* = 509	*n* = 555	*n* = 607	*n* = 777	*n* = 562
mean	31.3	32.9	35.3	37.1	40.7	39.2
min	15.0	16.6	15.0	16.0	15.5	16.0
max	61.0	78.0	77.7	80.7	95.0	87.0
median	29.0	31.0	33.5	35.9	39.0	36.0

**Fig. 1:**
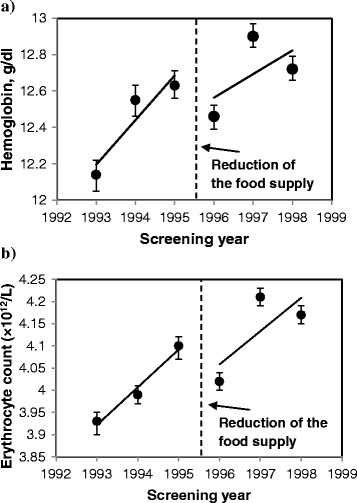
Adjusted mean (95 % CI) concentrations of hemoglobin (g/dl) (**a**) and erythrocyte counts (×10^12^/L) (**b**). In midyear 1995, the food supplementation at school was reduced from 3 to 2 meals per day. Linear models with repeated measures adjusted for food supplementation (2 meals/day vs. 3 meals/day), gender, age (continuous), interquartile range normalized ^137^Cs soil contamination levels in the area of residence and food × time interaction

In adjusted analyses, the mean levels of hemoglobin, erythrocytes and several other blood indices changed after the food intervention was reduced (Fig. 1, Additional file 1, Table 3). For example, when children received three meals with low content of artificial radionuclides per day, their mean hemoglobin levels had been steadily increasing from 12.14 g/dL (95 % CI: 12.05-12.22) in 1993 to 12.63 g/dL (95 % CI: 12.56-12.71) in 1995. After the food supplementation was reduced to two meals per day, the mean hemoglobin level in 1996 dropped to 12.46 g/dl (95 % CI: 12.39-12.52) and then started to increase again (Fig. 1a, Table 3). Similar changes were observed for erythrocytes and monocytes (Fig. 1b, Table 3, Additional file 1d). From 1995 to 1996, adjusted mean erythrocyte counts decreased from 4.10 × 10^12^/L (95 % CI: 4.07-4.12) to 4.02 × 10^12^/L (95 % CI: 4.00-4.04) and then they started to increase again (Fig. 1b, Table 3). Between 1995 and 1996, adjusted mean levels of leukocytes, neutrophils, platelets and neutrophil/lymphocyte ratio increased, the latter from 1.34 (95 % CI: 1.29-1.39) to 1.46 (95 % CI: 1.42-1.51) (Table 3, Additional file 1a, b, e, and h). Further, in 1996–1998 adjusted mean levels of platelets, leukocytes, lymphocytes, neutrophils and eosinophils kept increasing and exceeded their levels in 1993–1995 (Table 3, Additional file 1a-c, e, and f). Immunoglobulins A, G, and M concentrations increased from 1995 to 1996 and then had a downward trend (Table 3, Additional file 1j-l). The mean body mass index (BMI) kept increasing over time even after the food supply was reduced (Table 3). In the model, BMI was not associated with residential ^137^Cs soil levels (β = 0.09, *p*-value = 0.53).

**Table 3:** Relationships between school-based food supplementation and blood indices, 1993-1998

Blood index (and BMI)	Adjusted mean^a^ (95 % CI)					
	3 meals/d			2 meals/d		
	1993	1994	1995	1996	1997	1998
Hemoglobin (g/dL)	12.14 (12.05-12.22)	12.55 (12.46-12.63)	12.63 (12.56-12.71)	12.46 (12.39-12.52)	12.90 (12.84-12.97)	12.72 (12.66-12.79)
Erythrocytes (x10^12^/L)	3.93 (3.90-3.95)	3.99 (3.97-4.01)	4.10 (4.07-4.12)	4.02 (4.00-4.04)	4.21 (4.19-4.23)	4.17 (4.15-4.19)
Platelets (x10^9^/L)	225.88 (221.74-230.03)	246.12 (241.94-250.33)	241.12 (236.87-245.45)	254.47 (249.91-259.12)	278.86 (274.21-283.61)	273.88 (268.73-279.11)
Leukocytes (x10^9^/L)	5.14 (5.01-5.27)	5.82 (5.67-5.97)	6.31 (6.16-6.46)	6.76 (6.61-6.91)	7.32 (7.16-7.47)	7.24 (7.07-7.41)
Lymphocytes (x10^9^/L)	1.97 (1.92-2.03)	2.12 (2.06-2.19)	2.27 (2.20-2.33)	2.35 (2.29-2.40)	2.57 (2.51-2.63)	2.60 (2.53-2.67)
Monocytes (x10^9^/L)	0.30 (0.28-0.31)	0.42 (0.40-0.44)	0.52 (0.50-0.54)	0.49 (0.47-0.51)	0.61 (0.59-0.63)	0.60 (0.58-0.63)
Platelet/lymphocyte ratio	114.40 (110.85-118.07)	115.85 (112.10-119.73)	106.36 (103.24-109.56)	108.52 (105.57-111.53)	108.77 (105.97-111.62)	105.19 (102.04-108.43)
Neutrophil/lymphocyte ratio	1.27 (1.22-1.31)	1.32 (1.27-1.37)	1.34 (1.29-1.39)	1.46 (1.42-1.51)	1.39 (1.35-1.43)	1.32 (1.28-1.37)
Basophils (x10^9^/L)	0.049 (0.046-0.052)	0.064 (0.061-0.068)	0.062 (0.058-0.066)	0.059 (0.056-0.063)	0.078 (0.074-0.083)	0.077 (0.073-0.082)
Neutrophils (x10^9^/L)	2.50 (2.42-2.58)	2.80 (2.71-2.90)	3.03 (2.94-3.13)	3.43 (3.32-3.53)	3.56 (3.46-3.66)	3.43 (3.33-3.54)
Eosinophils (x10^9^L)	0.18 (0.17-0.19)	0.22 (0.21-0.24)	0.22 (0.21-0.24)	0.23 (0.22-0.24)	0.26 (0.24-0.27)	0.27 (0.26-0.29)
Immunoglobulin A (g/L)	0.71 (0.62-0.79)	0.78 (0.70-0.85)	0.88 (0.81-0.95)	0.96 (0.89-1.03)	0.95 (0.88-1.01)	0.91 (0.83-0.99)
Immunoglobulin G (g/L)	4.93 (4.35-5.51)	5.44 (4.94-5.94)	6.16 (5.70-6.62)	6.67 (6.22-7.12)	6.55 (6.12-6.97)	6.33 (5.82-6.85)
Immunoglobulin M (g/L)	0.52 (0.46-0.59)	0.62 (0.56-0.68)	0.69 (0.63-0.74)	0.77 (0.71-0.82)	0.75 (0.70-0.80)	0.72 (0.66-0.78)
BMI kg/m^2^	17.22 (16.99-17.44)	17.16 (16.96-17.36)	17.45 (17.27-17.63)	17.67 (17.50-17.83)	17.75 (17.60-17.89)	17.78 (17.61-17.94)

Detailed legend: From 1993 through the spring 1995 children received 3 radiation free meals at school. After the summer break, in the fall 1995, the food supplementation at school was reduced to 2 meals per day

^a^Linear models with repeated measures adjusted for the food supplementation (2 meals/day vs. 3 meals/day), gender, age (continuous), interquartile range normalized ^137^Cs soil contamination levels in the area of residence and food × time interaction. Generalized estimating equations (GEE) with exchangeable correlation structure were used to estimate parameters. The following blood indices were log-transformed for the analyses and back-transformed for presentation to yield geometric means and 95 % CI: basophils, eosinophils, leukocytes, lymphocytes, monocytes, neutrophils, platelets, platelet/lymphocyte ratio, and neutrophil/lymphocyte ratio

*CI:* Confidence interval

**Fig. 2:**
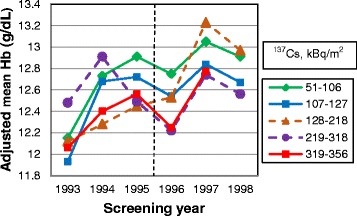
Adjusted mean hemoglobin concentrations (g/dL) by levels of residential ^137^Cs soil contamination. In midyear 1995, the food supplementation at school was reduced from 3 to 2 meals per day. Linear models with repeated measures adjusted for food supplementation (2 meals/day vs. 3 meals/day), gender, age (continuous), and food × time interaction. The analyses were stratified by levels of residential ^137^Cs soil contamination

The relationship between food supplementation and hemoglobin levels varied by level of residential ^137^Cs soil contamination (p_interaction_ < 0.0001) (Fig. 2). At the higher levels of ^137^Cs in soil the blood hemoglobin concentrations were lower. From 1993 to 1995, adjusted mean hemoglobin concentrations increased at all residential ^137^Cs levels except 219–318 kBq/m^2^. Between 1995 and 1996, when the food supplementation was reduced, mean hemoglobin concentrations decreased at all residential exposure levels except the middle level, 128–218 kBq/m^2^. Further, from 1996 to 1997, the mean hemoglobin concentrations at all ^137^Cs levels started to increase again followed by decline from 1997 to 1998.

**Fig. 3:**
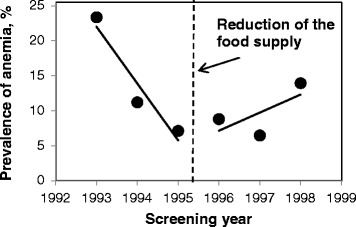
Prevalence of anemia in 1993–1995. In midyear 1995, the food supplementation at school was reduced from 3 to 2 meals per day. Log-binomial models with repeated measures adjusted for food (2 meals/d vs. 3 meals/d), gender, age (continuous), interquartile range normalized ^137^Cs soil contamination levels in the area of residence, and food × time interaction

**Fig. 4:**
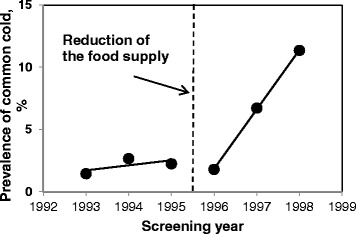
Prevalence of common cold in 1993–1995. In midyear 1995, the food supplementation at school was reduced from 3 to 2 meals per day. Log-binomial models with repeated measures adjusted for food (2 meals/d vs. 3 meals/d), gender, age (continuous), interquartile range normalized ^137^Cs soil contamination levels in the area of residence, and food × time interaction

**Fig. 5:**
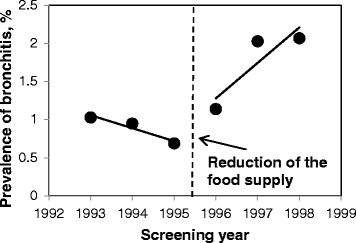
Prevalence of bronchitis in 1993–1995. In midyear 1995, the food supplementation at school was reduced from 3 to 2 meals per day. Log-binomial models with repeated measures adjusted for food (2 meals/d vs. 3 meals/d), gender, age (continuous), interquartile range normalized ^137^Cs soil contamination levels in the area of residence, and food × time interaction

Temporary changes in prevalence of various health conditions were plotted with trend lines to demonstrate discontinuity in the regressions (Figs. 3, 4, 5 and Additional file 2). Previously decreasing prevalence of anemia after 1995 started to increase (Fig. 3). Prevalence of common cold and bronchitis noticeably increased in 1996–1998 as compared to the preceding period of 1993–1995 (Figs. 4 and 5). Prevalence of chronic lymphadenitis had been overall increasing until 1995, and in 1996–1998 was decreasing (Additional file 2a). Prevalence of chronic inflammation of the tonsils and adenoids has been steadily decreasing from 1993 to 1998 with plateau between 1995 and 1996 (Additional file 2b). Allergy and atopic dermatitis were very rare in the study population (1-2 % and 0.5-1 %, respectively) and their prevalence only slightly increased over time (Additional file 2c, d). In adjusted analyses, the prevalence ratio of unspecified anemia had been decreasing by 43 % per year when children received 3 meals with low content of artificial radionuclides per day and increased by 31 % per year after the food supplementation was reduced (PR = 0.57, 95 % CI: 0.48-0.67 vs. PR = 1.3, 95 % CI: 1.11-1.57, p_interaction_ < .0001, Table 4). Prevalence of anemia in boys was 20 % lower than in girls but this difference was not statistically significant (PR _boys vs. girls_ = 0.80, 95 % CI: 0.64-1.01, *p* = 0.06). In stratified analyses (data not shown), prevalence ratio of anemia in girls was 40 % lower in the years between 1993 and 1995 (PR = 0.60, 95 % CI: 0.49-0.73) and between 1995 and 1998 it was increasing by 38 % per year (PR = 1.38, 95 % CI: 1.09-1.75). In boys, the prevalence ratio of anemia in 1993–1995 was 49 % lower (PR = 0.51, 95 % CI: 0.39-0.68), and in the years 1995–1998 it increased to 22 % (PR = 1.22, 95 % CI: 0.93-1.60). Also, the prevalence ratio of common cold was higher after the reduction of food supplementation as compared to the prior period (PR = 1.27, 95 % CI: 0.87-1.84 vs. 2.32, 95 % CI: 1.79-3.00, p_interaction_ = 0.01; Table 4). Similarly, prevalence ratio of bronchitis also increased from 1.09 (95 % CI: 0.81-1.48) to 1.24 (95 % CI: 0.81-1.90, p_interaction_ = 0.43) per year.

**Table 4:** Prevalence of various health conditions before and after the reduction of the school-based food supplementation

Health condition^a^	1993-1995		1996-1998		Food × time interaction, *p*-value^d^
	3 meals/d		2 meals/d		
	Intercept^b^	PR^c^ (95 % CI)	Intercept^b^	PR^c^ (95 % CI)	
Unspecified anemia	0.64	0.57 (0.48-0.67)	0.05	1.31 (1.11-1.57)	<.0001
Allergy^e^	0.01	1.41 (0.84-1.93)	0.01	1.26 (0.82-1.93)	0.72
Atopic dermatitis	0.005	1.22 (0.69-2.14)	0.01	1.02 (0.58-1.82)	0.52
Bronchitis	0.02	1.09 (0.81-1.48)	0.02	1.24 (0.81-1.90)	0.43
Common cold	0.07	1.27 (0.87-1.84)	0.01	2.32 (1.79-3.00)	0.01
Lymph node enlargement	0.73	1.01 (0.92-1.11)	0.46	1.07 (0.93-2.23)	0.49
Chronic tonsillitis/adenoiditis	0.23	0.91 (0.86-0.96)	0.22	0.93 (0.84-1.03)	0.52

Detailed legend: From 1993 through the spring 1995 children received 3 radiation free meals at school. After the summer break, in the fall 1995, the food supplementation at school was reduced to 2 meals per day

^a^As specified by ICD-9

^b^Intercept is a prevalence ratio of a disease at the threshold of food supplementation (midyear 1995). A difference between intercepts for the full and reduced food supplementation periods demonstrates regression discontinuity

^c^Log-binomial models with repeated measures adjusted for food (2 meals/d vs. 3 meals/d), gender, age (continuous), interquartile range normalized ^137^Cs soil contamination levels in the area of residence, and food × time interaction. Generalized estimating equations (GEE) with exchangeable correlation structure were used to estimate parameters

^d^Wald test

^e^Includes: allergic rhinitis, atopic dermatitis, unspecified allergy and related conditions

CI: Confidence interval; PR: Prevalence ratio

## Discussion

After the reduction of the school-based food supplementation the rates of improvement of several hematological markers, including hemoglobin, erythrocytes, leukocytes, monocytes, neutrophils and neutrophil/lymphocyte ratio observed in previous years decreased. Between 1995 and 1996, concentrations of hemoglobin and erythrocytes significantly decreased and blood counts of neutrophils, platelets, neutrophil/lymphocyte ratio, and serum levels of immunoglobulins A, G and M, on the contrary, increased. In agreement, after the food supplementation was reduced, in 1996–1998, prevalence of anemia, common cold and bronchitis increased. We also found that the relationship between reduction of the number of meals with low content of artificial radionuclides and levels of hemoglobin was modified by levels of residential ^137^Cs soil contamination. Mean increase in hemoglobin levels before and after the reduction of food supplementation was lower at the higher ^137^Cs soil levels.

One of potential limitations of this study is its quasi-experimental nature. The number of meals with low content of artificial radionuclides (3 vs. 2) per day was not randomly assigned with the purpose to investigate its effects, and analyses were performed retrospectively. However, quasi-experimental evaluations of public health interventions are essential when randomized controlled trials are not possible or ethical. The main concern with such designs is potential confounding from contemporaneous influential events [32, 33]. What other population-level changes may have coincided with the temporal change of interest? Furthermore, retrospective analysis of health surveillance data may include biases due to the fact that the data are not collected for the purpose of evaluating interventions and/or research. No additional data were collected on other environmental and/or nutritional risk factors coinciding with the radiation exposure that could be employed in this analysis. However, it is unlikely that there were any population-level changes between 1993–1995 and 1996–1998 other than the reduction in food supplementation. The regression discontinuity analysis combined with the additional adjustment for variables that were unbalanced at the threshold (age, gender and ^137^Cs soil levels) makes this study similar in rigor to a randomized controlled trial; thus, near the threshold all temporally invariant confounding is adjusted for by design.

Another limitation of this analysis is that in the statistical models we adjusted for residential ^137^Cs contamination levels as a proxy of individual (internal and external) exposure which could have introduced its misclassification. This proxy measure doesn't take into account individual food intake and adherence to protective recommendations. The data on ^137^Cs levels in food consumed at home were not collected in this study. The magnitude of the associated error, however, would not be strong since the individual ^137^Cs activity in a body was positively correlated with residential soil contamination levels in previous studies [6, 13, 34] and in this study as well.

The improvement of blood indices seen from 1993 to 1995 was most likely due to the natural weathering and decay of cesium in the environment and the countermeasures introduced by the Ukrainian government aiming to reduce the internal exposures to radionuclides. These countermeasures included prohibition of farming in highly contaminated areas, warning against consumption of forest goods and game, decontamination of locally grown produce, adding cesium binders to cattle foods and importation of food with low content of artificial radionuclides from other areas [14, 15, 35]. In some areas of Ukraine, these countermeasures have been shown to reduce individual doses from 40 % up to 4-fold [35]. Substitution of locally produced food by food with low content of artificial radionuclides imported from other areas reduced internal dose in adults residing in rural areas by 70-86 % [36]. Thus, it is highly likely that provision of food with low content of artificial radionuclides at schools in the Ukraine significantly reduced children's internal exposure to ^137^Cs. When reducing this food supplementation, the internal radiation exposure potentially increased due to the consumption of the contaminated food at home. A prior study has shown that the individual radiation doses in Narodichi region increased up to 2–3 times from May through October due to consumption of locally grown produce and forest goods [37]. Therefore, the differences in time trends may be attributed to the higher internal ^137^Cs exposure after 1995. Our finding that the mean hemoglobin levels during both periods of food supplementation (1993–1995 and 1996–1998) were lower at the higher levels of residential ^137^Cs soil contamination also suggests that ingestion of ^137^Cs from contaminated food was likely the main driving force of changes in hematologic parameters. It is likely that changes in blood indices were underestimated since all health examinations and blood tests were conducted in the spring, when the main sources of internal exposure are milk, meat and potatoes; thus, they did not capture the changes due to higher exposures from locally grown foods and forest goods consumed during summer and fall [37]. Few studies exist on the effects of low-dose chronic exposure to radiation in children. In the previous reported research, the relationship between residential soil contamination with ^137^Cs and measured hematologic parameters was investigated [22]. The results indicated persistent adverse hematological effects associated with ^137^Cs exposure. The blood cell counts were reduced with increasing soil contamination. In the observation period all parameters did improve, partly attributed to the natural weathering and decay of ^137^Cs in the environment and the regenerative capacity of the hematopoietic system. However, the cessation of food supplementation was not considered at that time within the study design. In this study, after the food supplementation was reduced in midyear 1995, the hemoglobin concentrations and erythrocyte counts in 1996 immediately dropped and the temporal improvement in hemoglobin levels in the following years decreased, which was corroborated by an increase in diagnoses of anemia. Three factors could potentially cause anemia in this population: increased consumption of radionuclides from locally produced food, poor nutrition (if nutritional value of locally grown food is lower compared to the imported food), and increasing proportion of girls who reached menarche. According to previous studies, even before the Chernobyl nuclear incident the food consumed at home lacked proteins, sufficient vitamins and minerals, and provided excessive amounts of refined carbohydrates and fat [11]. In this study we were unable to compare the nutrient content of food consumed at home and at school since the information about diet at the individual level was not collected. Large individual variability in nutrient content of food consumed at home seems unlikely since the population consists of poor villagers with similar socio-economic status and food access. In this study, the mean BMI kept increasing even after the food supplementation was reduced, thus, arguing against decreased quality of nutrition as a driving factor for increased prevalence of anemia. Further studies are needed to confirm this conclusion. Despite adjustment for gender and age, possibility of residual confounding by these factors, however, cannot be completely ruled out. Between 1995 and 1996 the proportion of thirteen-year old girls (the average age of menarche in Ukraine) in the study sample decreased from 9 % to 8 %; however, the overall proportion of girls 13 years of age and older increased by 5 % and continued to increase in the following years due to cohort aging. The sex-stratified analyses showed that prevalence of anemia in the years 1993–1995 was decreasing in both boys and girls and in 1995–1998 it was increasing thereafter in both girls and boys. The results suggest that the anemia was slightly more prevalent among girls than among boys; however, this difference was not statistically significant.

Various observed changes in levels of white blood cells and concentrations of serum IgA, IgG and IgM after the food supplementation was reduced indicate that increased exposure to ^137^Cs ingested with food affected the immune system. Altered immune reactivity may lead to increased susceptibility to infections and respiratory pathology in this population, as was previously described [23]. We found that the prevalence of common cold and bronchitis significantly increased after the food supplementation was reduced.

No studies are available to date that document the effect of a school-based food intervention aimed at the reduction of internal radiation exposure due to contaminated food. Another more recent large-scale public health intervention occurred that targeted all age groups. After the large nuclear incident that occurred on March 11, 2011 in Fukushima, Japan, the Japanese government introduced monitoring of contamination of tap water and foodstuffs for various radionuclides, including ^131^I, ^134^Cs and ^137^Cs. The distribution and consumption of foodstuffs with levels of contamination exceeding provisional regulation values were restricted, and infants in Tokyo were provided clean bottled water without cost [38]. However, unlike after the Chernobyl disaster, Japanese children were not provided with food supplementation regulated by nutritional standards, and many of them experienced food shortages [39]. The effects of internal exposure to radionuclides from contaminated food on children's health in Japan have not yet been well described, and the length of follow up is currently much shorter than after the Chernobyl disaster.

## Conclusions

The food with low content of artificial radionuclides provided for children by the Ukrainian government following the Chernobyl disaster likely prevented the development of anemia in many of the children residing in the contaminated districts. Food supplementation reduction was associated with increased prevalence of anemia and acute respiratory illnesses. Further research is needed over a longer period of time to forecast the concentrations of hemoglobin and other hematological measurements to better estimate the impact of this public health intervention and see if increasing food supplementation would be beneficial at this point in time after the accident. Also, measurement of whole-body radiation burden would improve future study designs.

## Additional files

Additional file 1:
**Adjusted mean blood indices in 1993–1995.** a) Adjusted mean (95 % CI) blood leukocyte count (×109/L). b) Adjusted mean (95 % CI) blood neutrophil count (×10^9^/L). c) Adjusted mean (95%CI) blood lymphocyte count (×10^9^/L). d) Adjusted mean (95 % CI) blood monocyte count (×10^9^/L). e) Adjusted mean (95 % CI) blood platelet count (×10^9^/L). f) Adjusted mean (95 % CI) blood eosinophil count (×10^9^/L). g) Adjusted mean (95 % CI) blood basophil count (×10^9^/L). h) Adjusted mean (95 % CI) blood neutrophil/lymphocyte ratio. i) Adjusted mean (95 % CI) blood platelet/lymphocyte ratio. j) Adjusted mean (95 % CI) serum IgA concentration (g/L). k) Adjusted mean (95 % CI) serum IgG concentration (g/L). l) Adjusted mean (95 % CI) serum IgM concentration (g/L). (PDF 244 kb) Additional file 2:
**Prevalence of various diseases in 1993–1995.** a) Chronic lymphadenitis. b) Chronic tonsillitis or hypertrophy of tonsils with adenoids. c) Allergy. d) Atopic dermatitis. (PDF 97 kb)

## Abbreviations

^137^Cs: ^137^Cesium
CI: Confidence interval
EDTA: Ethylenediaminetetraacetic acid
GEE: Generalized Estimating Equations
Ig: Immunoglobulin
NCC: Narodichi Children Cohort
