# Supplementary material for: Dietary supplementation with radionuclide free food improves children's health following community exposure to 137Cesium: a prospective study
Source: Environ Health. 2015 Dec 22;14:94. doi: 10.1186/s12940-015-0084-x (PMC4687105; doi:10.1186/s12940-015-0084-x)

Additional file 1. Adjusted mean blood indices in 1993-1995. In midyear 1995, the food supplementation at school was reduced from 3 to 2 meals per day. Linear models with repeated measures adjusted for food supplementation (2 meals/day vs. 3 meals/day), gender, age (continuous), interquartile range normalized ^{137}Cs soil contamination levels in the area of residence and food×time interaction**.**

a) Adjusted mean (95%CI) blood leukocyte count (×10^9/L)

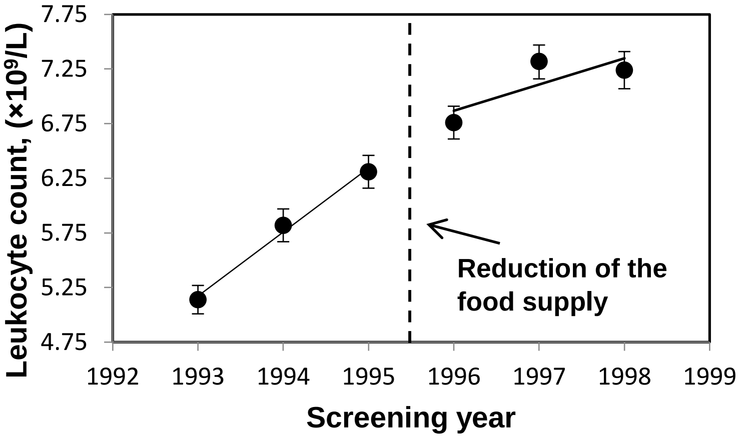

b) Adjusted mean (95%CI) blood neutrophil count (×10^9/L)

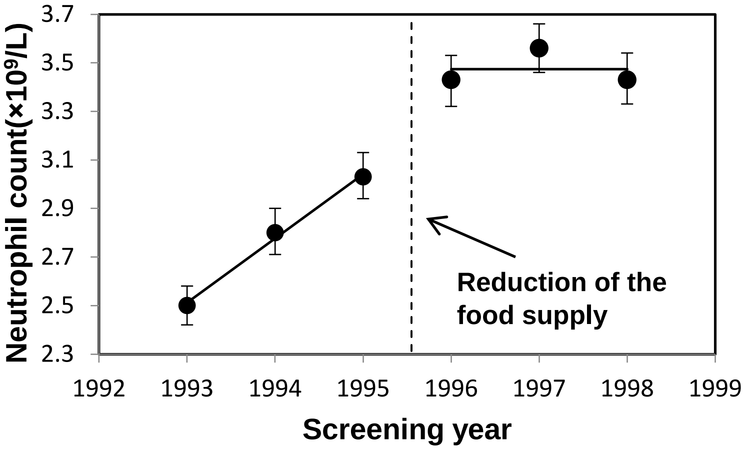

c) Adjusted mean (95%CI) blood lymphocyte count (×10^9/L)

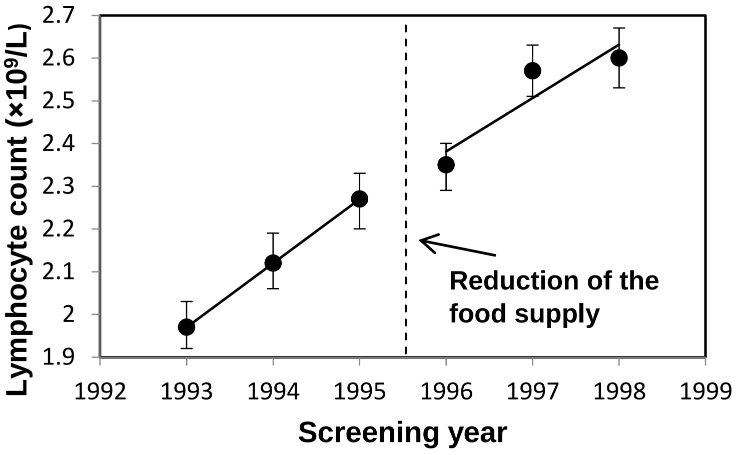

d) Adjusted mean (95%CI) blood monocyte count (×10^9/L)

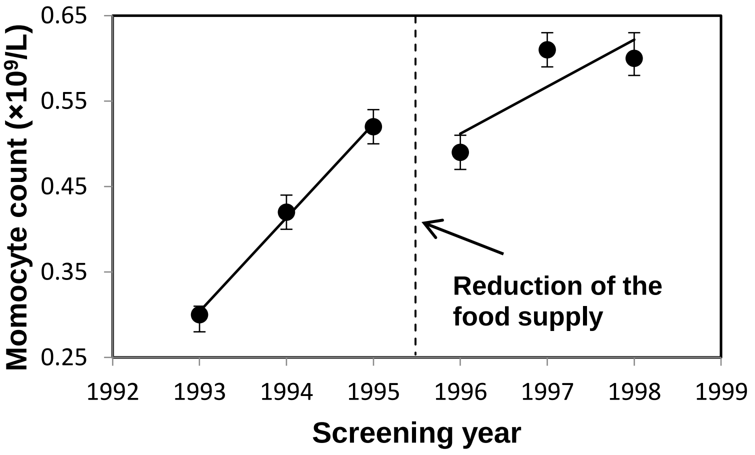

e) Adjusted mean (95%CI) blood platelet count (×10^9/L)

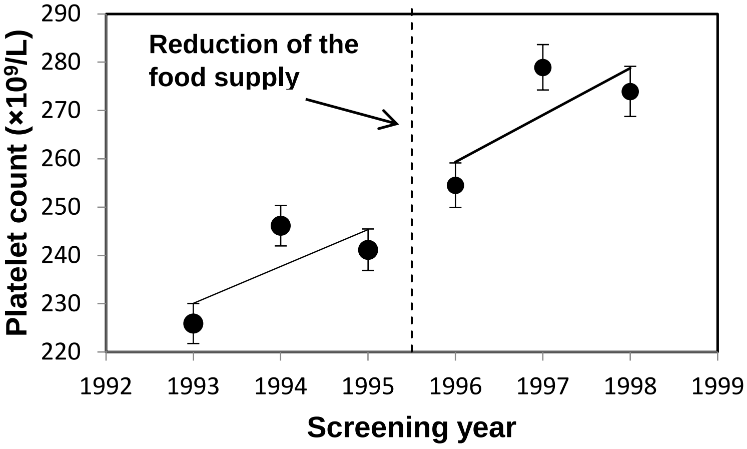

f) Adjusted mean (95%CI) blood eosinophil count (×10^9/L)

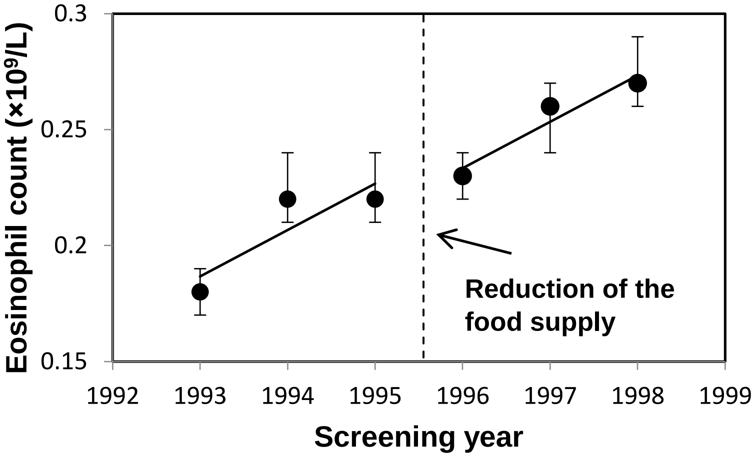

g) Adjusted mean (95%CI) blood basophil count (×10^9/L)

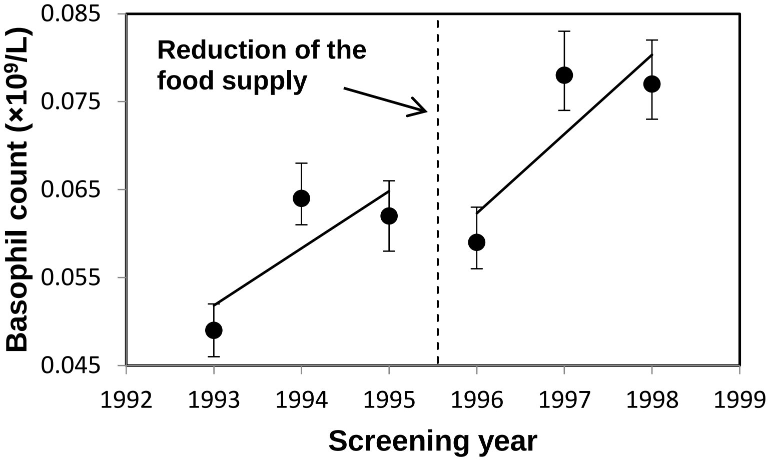

h) Adjusted mean (95%CI) blood neutrophil/lymphocyte ratio

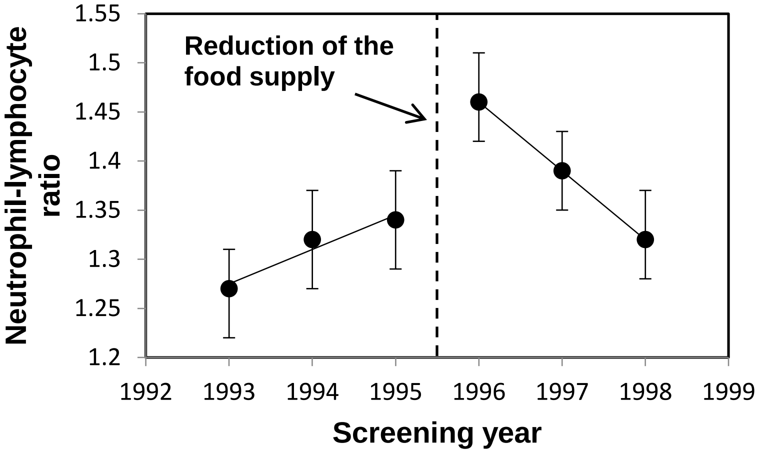

i) Adjusted mean (95%CI) blood platelet/lymphocyte ratio

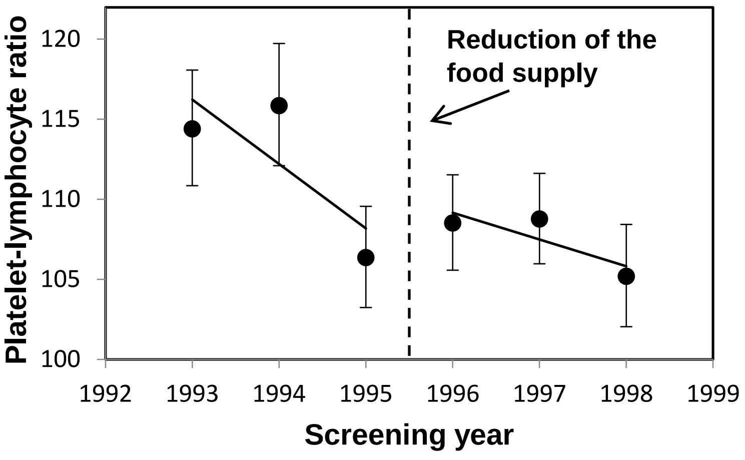

j) Adjusted mean (95%CI) serum IgA concentration (g/L)

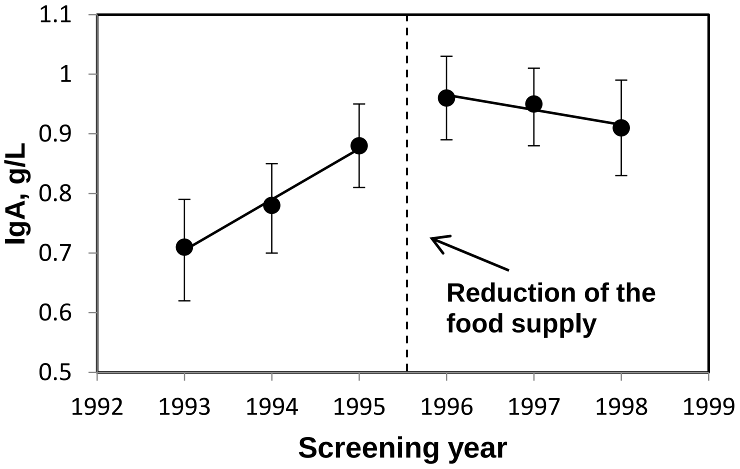

k) Adjusted mean (95%CI) serum IgG concentration (g/L)

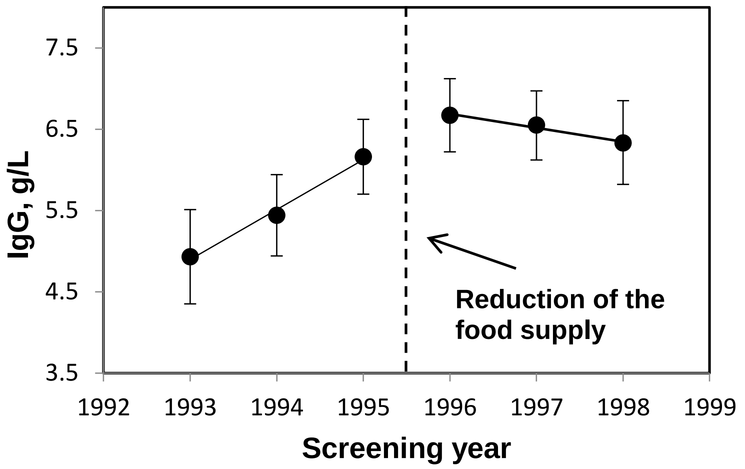

l) Adjusted mean (95%CI) serum IgM concentration (g/L)

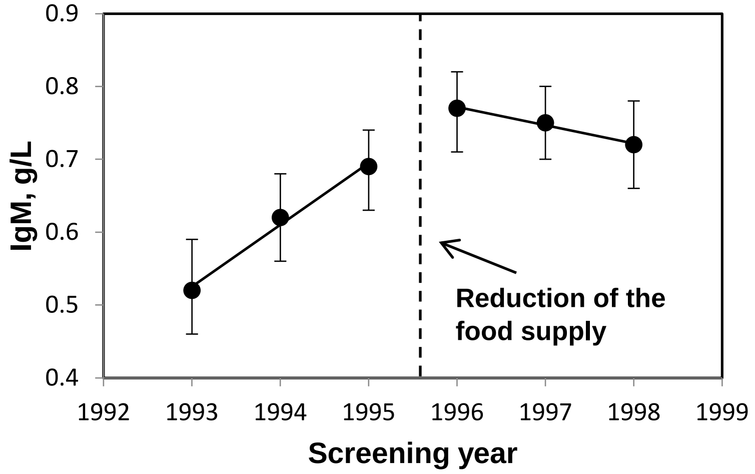

Supplement: Additional file 1: — Adjusted mean blood indices in 1993–1995. a) Adjusted mean (95 % CI) blood leukocyte count (×109/L). b) Adjusted mean (95 % CI) blood neutrophil count (×109/L). c) Adjusted mean (95%CI) blood lymphocyte count (×109/L). d) Adjusted mean (95 % CI) blood monocyte count (×109/L). e) Adjusted mean (95 % CI) blood platelet count (×109/L). f) Adjusted mean (95 % CI) blood eosinophil count (×109/L). g) Adjusted mean (95 % CI) blood basophil count (×109/L). h) Adjusted mean (95 % CI) blood neutrophil/lymphocyte ratio. i) Adjusted mean (95 % CI) blood platelet/lymphocyte ratio. j) Adjusted mean (95 % CI) serum IgA concentration (g/L). k) Adjusted mean (95 % CI) serum IgG concentration (g/L). l) Adjusted mean (95 % CI) serum IgM concentration (g/L). (PDF 244 kb) [file 12940_2015_84_MOESM1_ESM.pdf]
