# Supplementary material for: Dietary supplementation with radionuclide free food improves children's health following community exposure to 137Cesium: a prospective study
Source: Environ Health. 2015 Dec 22;14:94. doi: 10.1186/s12940-015-0084-x (PMC4687105; doi:10.1186/s12940-015-0084-x)

Additional file 2. Prevalence of various diseases in 1993-1995. In midyear 1995, the food supplementation at school was reduced from 3 to 2 meals per day. Log-binomial models with repeated measures adjusted for food (2 meals/d vs. 3 meals/d), gender, age (continuous), interquartile range normalized ^{137}Cs soil contamination levels in the area of residence, and food×time interaction.

a) Chronic lymphadenitis

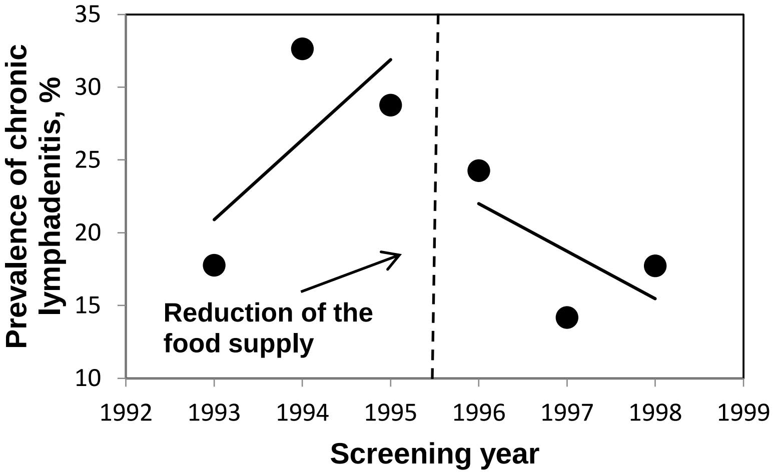

b) Chronic tonsillitis or hypertrophy of tonsils with adenoids

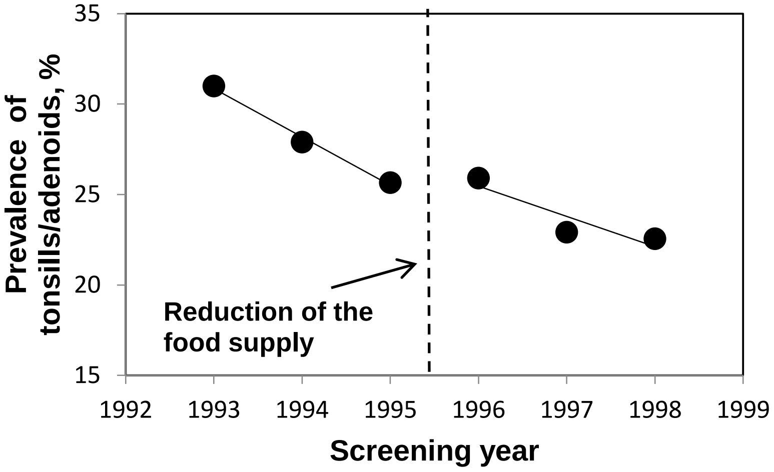

c) Allergy

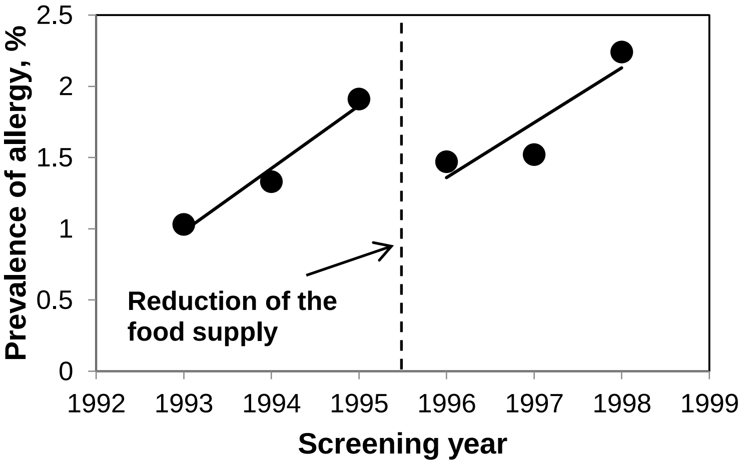

d) Atopic dermatitis

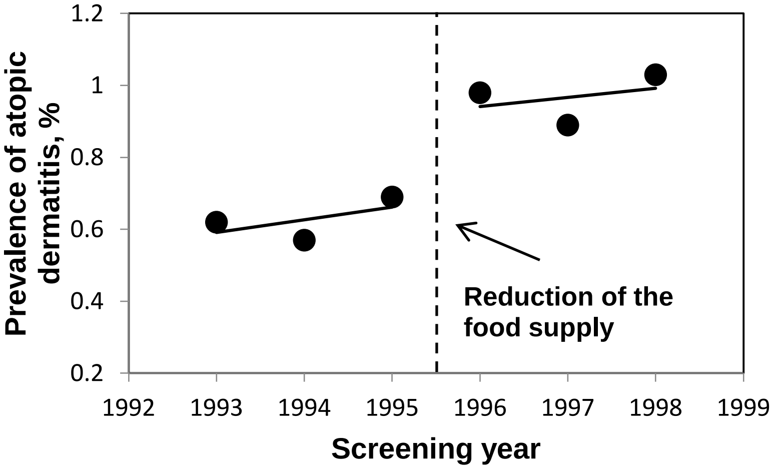

Supplement: Additional file 2: — Prevalence of various diseases in 1993–1995. a) Chronic lymphadenitis. b) Chronic tonsillitis or hypertrophy of tonsils with adenoids. c) Allergy. d) Atopic dermatitis. (PDF 97 kb) [file 12940_2015_84_MOESM2_ESM.pdf]
